# Formal health care costs among older people in Ireland: methods and estimates using The Irish Longitudinal Study on Ageing (TILDA)

**DOI:** 10.12688/hrbopenres.13692.1

**Published:** 2023-03-04

**Authors:** Peter May, Frank Moriarty, Eimir Hurley, Soraya Matthews, Anne Nolan, Mark Ward, Bridget Johnston, Lorna Roe, Charles Normand, Rose Anne Kenny, Samantha Smith

**Affiliations:** 1 Cicely Saunders Institute of Palliative Care, Policy & Rehabilitation, King's College London, London, UK; 2 Centre for Health Policy and Management, School of Medicine, Trinity College Dublin, Dublin, Ireland; 3 The Irish Longitudinal Study on Ageing, School of Medicine, Trinity College Dublin, Dublin, Ireland; 4 School of Pharmacy and Biomolecular Sciences, Royal College of Surgeons in Ireland, Dublin, Ireland; 5 Economic and Social Research Institute, Dublin, Ireland

**Keywords:** costs, ageing, demography, policy, functional limitations, end of life, proximity to death

## Abstract

**Background:** Reliable data on health care costs in Ireland are essential to support planning and evaluation of services. New unit costs and high-quality utilisation data offer the opportunity to estimate individual-level costs for research and policy.

**Methods:** Our main dataset was The Irish Longitudinal Study on Ageing (TILDA). We used participant interviews with those aged 55+ years in Wave 5 (2018) and all end-of-life interviews (EOLI) to February 2020. We weighted observations by age, sex and last year of life at the population level. We estimated total formal health care costs by combining reported usage in TILDA with unit costs (non-acute care) and public payer reimbursement data (acute hospital admissions, medications). All costs were adjusted for inflation to 2022, the year of analysis. We examined distribution of estimates across the population, and the composition of costs across categories of care, using descriptive statistics. We identified factors associated with total costs using generalised linear models.

**Results:** There were 5,105 Wave 5 observations, equivalent at the population level to 1,207,660 people aged 55+ years and not in the last year of life, and 763 EOLI observations, equivalent to 28,466 people aged 55+ years in the last year of life. Mean formal health care costs in the weighted sample were EUR 8,053; EUR 6,624 not in the last year of life and EUR 68,654 in the last year of life. Overall, 90% of health care costs were accounted for by 20% of users. Multiple functional limitations and proximity to death were the largest predictors of costs. Other factors that were associated with outcome included educational attainment, entitlements to subsidised care and serious chronic diseases.

**Conclusions:** Understanding the patterns of costs, and the factors associated with very high costs for some individuals, can inform efforts to improve patient experiences and optimise resource allocation.

## Introduction

### Background

Health care spending accounted for approximately 9% of gross domestic product in Organisation for Economic Co-Operation and Development (OECD) countries in 2019, the last complete year before the COVID-19 pandemic ^1^. Resource allocation decisions in health care therefore have substantial impacts at the macroeconomic level, but also at the microeconomic level, where funding and availability of services may affect individual health, wealth and productivity ^2^.

Health-related demands will always exceed available resources, placing a moral and practical imperative on decision-makers to fund those services that provide the best value ^3^. This challenge, which economists frame in terms of ‘scarcity’, will be increasingly complex through the 21 ^st^ century as populations age. Research has repeatedly shown that at the individual level the most important drivers of rising costs are not age *per se*, but instead, on the demand side, proximity to death, and, on the supply side, technology and staffing ^4, 5^. At the population level, costs will increase due to the rising total number of people living and dying with serious medical illness ^6^, the demand for health care workers growing more quickly than supply ^7, 8^, and the number of medical technologies increasing persistently ^5, 9– 11^.

Ireland is early in the demographic ageing process compared to other high-income countries ^12^, but faces the same structural challenges and the same need to reform health care services for the population health needs and resource constraints of the 21 ^st^ century ^13, 14^. A relatively young population today translates to faster-growing future needs: Ireland has the fastest ageing population in the European Union, with an expected three-fold increase in those aged 80+ years and near doubling of annual deaths in the next 20 years ^15^. Compared to other western European nations, the Irish health care system has long been distinguished by a combination of relatively high per-capita spending and a relatively limited basket of entitlements under universal coverage ^16^. Notable system characteristics include a reliance on acute inpatient hospital admissions given weak primary care capacity, high medications spending, non-compulsory insurance that improves access to some services for policyholders, and fast-growing population health needs ^16– 18^. Partly in recognition of these issues, Ireland has engaged in a wide-ranging health policy update since 2017, known as the ‘Sláintecare’ reforms, with mixed progress ^19– 22^.

### Context, rationale and aims

High-quality data on individual-level health care costs in Ireland are essential to support monitoring, planning and evaluation of services, and the allocation of scarce resources to maximise public welfare. The lack of a unique patient identifier prevents researchers from using routine administrative data to estimate individual-level costs ^23^. In 2021 researchers published the first standardised set of unit costs for non-acute health care in Ireland ^24^, a long-awaited development and a critical step for health economics research in the state ^25^.

The Irish Longitudinal Study on Ageing (TILDA) is a biennial study of people aged 50+ years in the Republic of Ireland that started in 2009–2011. Among many individual-level variables in a rich dataset, TILDA collects data on participants’ demographic, socioeconomic, early life, physical and mental health, and household characteristics ^26^. Utilisation data are collected via frequency questions on categories of health, social and residential care in the preceding year, *e.g.*, ‘how many times did you visit the GP in the last 12 months?’ While previous papers have estimated costs in TILDA ^27, 28^, these have been constrained by incomplete availability of unit costs in non-acute care and crude casemix estimates for acute care.

Combining the new unit cost database with TILDA offers the opportunity to estimate in the greatest detail yet health, social and residential care costs (henceforth, ‘health care costs’) among a population-representative sample of older people in Ireland. We supplement this new non-acute unit cost database with our own analyses of hospital inpatient admissions, adjusting for age, sex, diagnoses and discharge status, and a costing exercise of medications reported by TILDA respondents. The arising estimates can inform ongoing research studies, including those evaluating specific policies and models of delivery ^29^, delineating patterns and trajectories of health care use ^30^, surveying end-of-life needs ^31, 32^, and projecting future needs and costs ^33– 35^. They can also contribute to future studies both within TILDA, for example prediction exercises to identify high-cost users ^27^; and in wider modelling frameworks, for example cost-effectiveness analyses that have to characterise disease trajectories and costs in different clinical populations ^36^.

Our aims in this paper are to first document the methods by which health care costs are estimated in TILDA, and then to address the following research questions:

1. What are the health care costs for older people in Ireland? How are costs distributed across the population?
2. What is the underlying composition of these costs between primary and community care, hospital care, home care, residential care, and medications?
3. What individual-level predictors are associated health care costs?

## Methods

### Study design, participants and data

This is a costing study using secondary data sources. Our main data source was TILDA, which recruited a population-representative sample of more than 8,000 community-dwelling people aged 50+ years at Wave 1 (2009–2011) ^37^. Full details of the study design, recruitment, consent and data collection are available elsewhere ^26^. Briefly, computer-assisted personal interview (CAPI) and a self-completion questionnaire (SCQ) are used to collect data on demographic and socioeconomic characteristics such as early life, household composition, employment history, income and asset levels, as well as detailed information on health status ( *e.g.*, diagnoses, functional status, self-reported physical and mental health) and healthcare utilisation. When a participant dies, a family member or close friend is approached to conduct a voluntary end-of-life interview (EOLI) on the decedent’s experiences in the last 12 months of life. This process, including the ethical guidelines and procedures, has been detailed elsewhere ^28^. The EOLI represents a shortened version of the CAPI, asking the respondent questions on the decedent’s living situation, health, health care use and other factors. 

The baseline sample were invited to participate in CAPI and SCQ follow-up at Wave 2 (2012), Wave 3 (2014), Wave 4 (2016), Wave 5 (2018) and Wave 6 (2021, delayed from 2020 by the COVID-19 pandemic). Ethical approval for each wave is obtained from the Faculty of Health Sciences Research Ethics Committee in Trinity College Dublin. Participants make an informed decision about their participation, receiving advanced notice and information booklets; they may refuse to take part in any study section or withdraw at any time without justification; for each CAPI and EOLI question, available answers include “Refuse to answer” and “Don’t know”.

Secondary data sources were two unit cost databases for non-acute costs ^24, 38^, and the Hospital Inpatient Enquiry (HIPE) database of admissions to public acute hospitals ^39^. We also draw on Census data from the Central Statistics Office (CSO), for the purposes of population weighting, and the General Register Office (GRO) to identify deaths ^40^. All deaths in the Republic of Ireland must be recorded with the GRO, and TILDA is linked to the GRO in a process described previously ^41^.

In terms of perspective, we estimate the cost associated with providing the formal health care that TILDA participants and EOLI respondents report. We do not analyse or report out-of-pocket spending, which has implications for how costs are distributed between the system and households, and we do not analyse or report unpaid care provided by family and friends.

### Variables

***Dependent variable***

The primary dependent variable is formal health care costs, which combines the estimated costs associated with acute and non-acute care, and medications, reported by participants.

For acute and non-acute care, TILDA collects data on the frequency ( *f*) of use for a number ( *n*) of categories ( *h*), where *n* varies slightly between CAPI and EOLI, because hospice inpatient stays are not asked in the CAPI (implicitly assumed as zero). A unit cost ( *c*) was identified for each category. Therefore, for each individual CAPI or EOLI ( *i*), a specific acute or non-acute category ( *h*) has associated costs ( *y _i,h_*) given by:


$$y_{i,h}=f_{i,h}*c_{h}$$

Unit costs for non-acute care have been calculated in two prior costing exercises by Brick *et al.* ^38^ and Smith *et al.* ^24^ Hospital emergency department and outpatient unit costs have been calculated previously by Keegan *et al.* ^34^ We were not aware of any unit costing exercise for acute inpatient admissions that was coherently linkable with individual TILDA data, and so we calculated acute inpatient unit costs using HIPE data in a procedure detailed in ‘Appendix 1’, which can be found as *Extended data* ^42^. Briefly, in TILDA, we categorised each CAPI and/or EOLI to a category based on age, sex and diagnostic profile. In HIPE, we calculated the reimbursement due for each overnight adult admission to a public acute hospital in Ireland between 2009 and 2019 using the Healthcare Pricing Office (HPO) activity-based funding (ABF) guidance ^43^, which determines reimbursement rates according to primary diagnosis and length of stay. We categorised each of these admissions by age, sex and diagnostic profile, and then calculated acute unit costs for each age/sex/diagnostic group as the mean reimbursement for an overnight stay in that group. We linked these acute unit costs to each CAPI and EOLI by age/sex/diagnostic profile, incorporating for EOLIs the additional cost associated with a death in hospital.

In all cases we chose the most recently available unit costs available. These most recent unit costs were calculated in different years. We standardised all unit costs to 2022, the year that the analyses were conducted, using the Consumer Price Index (CPI) for health ^44, 45^. In data processing we created sub-groups for ease of interpretation: primary and community care, hospital care, home care, residential care, and medications. Each category of care, its’ variable name in the most recent publicly available CAPI and EOLI, the unit cost source, the unit cost after adjusting to 2022 prices, and the sub-group to which it was allocated are presented in Table 1.

**Table 1.** Unit costs for categories of health care use collected in the CAPI and EOLI.

Sub-Group	Category	Unit cost source	Unit cost (2022 EUR)	CAPI ^48^	EOLI ^§^
***Hospital***	Emergency department	Keegan *et al.* ^34 (i)^	EUR 321 per visit	hu007	xt_hu010
	Outpatient visit	Keegan *et al.* ^34 (i)^	EUR 184 per visit	hu008	xt_hu011
	Overnight inpatient admits	*Authors’ own* ^(ii)^	*By age/sex/dx*	hu010	xt_hu013 ^(iii)^ xt_cs021 ^(iv)^
***Primary and community***	General Practitioner	Smith *et al.* ^24 (v)^	EUR 49 per visit	hu005	xt_hu005
	Public Health Nurse	Smith *et al.* ^24 (v)^	EUR 60 per visit	hu015_01 ^(vi)^	xt_hu029_01
	Occupational therapist	Smith *et al.* ^24 (v)^	EUR 69 per visit	hu015_02 ^(vi)^	xt_hu029_02
	Chiropodist	Smith *et al.* ^24 (v)^	EUR 69 per visit	hu015_03 ^(vi)^	xt_hu029_03
	Physiotherapist	Smith *et al.* ^24 (v)^	EUR 69 per visit	hu015_04 ^(vi)^	xt_hu029_04
	Speech & lang. therapist	Smith *et al.* ^24 (v)^	EUR 69 per visit	hu015_05 ^(vi)^	xt_hu029_05
	Social worker	Smith *et al.* ^24 (v)^	EUR 47 per visit	hu015_06 ^(vi)^	xt_hu029_06
	Psychologist	Smith *et al.* ^24 (v)^	EUR 106 per visit	hu015_07 ^(vi)^	xt_hu029_07
	Day care	Brick *et al.* ^38 (vii)^	EUR 48 per visit	hu015_11 ^(vi)^	xt_hu029_08
	Dentist	Smith *et al.* ^24 (v)^	EUR 35 per visit	hu015_13 ^(vi)^	xt_hu029_10
	Dietician	Smith *et al.* ^24 (v)^	EUR 69 per visit	hu015_15 ^(vi)^	xt_hu029_12
***Home***	Home help ^(xiii)^	Smith *et al.* ^24 (v)^	EUR 35 per hour	hu015A	xt_hu022
	Personal care attendant ^(ix)^	Smith *et al.* ^24 (v)^	EUR 36 per hour	hu015B	xt_hu025
	Meals on wheels	Brick *et al.* ^38 (vii)^	EUR 12 per visit	hu015C	xt_hu027
	Home care package ^(ix)^	Smith *et al.* ^24 (v)^	EUR 36 per hour	hu015D	xt_hu074
***Residential***	Nursing home ^(x)^	Smith *et al.* ^24 (v)^	EUR 1,722 per week	hu032	xt_cs025
	Hospice	Brick *et al.* ^38 (vii)^	EUR 999 per night	n/a ^(xi)^	xt_cs023 ^(iv)^

^§^ EOLIs are not published on the study website (tilda.tcd.ie), but access may be applied for at that location. ^(i)^ Keegan *et al.*, estimated costs for 2018; per the CSO CPI Health, the multiplier from December 2018 to December 2022 was 1.076. ^(ii)^ Overnight admissions were costed using HIPE, detailed ‘Appendix 1’ in *Extended data* ^42^. ^(iii)^ Where EOLI reports death in hospital, the unit cost for that admission is adjusted (see Appendix 1) ^42^. ^(iv)^ Where EOLI reports people admitted to hospital or hospice as an inpatient, these episodes were costed using the relevant category unit cost and reported under that sub-group; where EOLI reports a decedent was living in a hospital or hospice as their main residence, these episodes were costed using the nursing home unit cost and reported under the sub-group ‘residential care’. ^(v)^ Smith *et al.*, estimated costs for 2019; per the CSO CPI Health, the multiplier from December 2019 to December 2022 was 1.066. Smith *et al.*, report different scenarios, we use the baseline public system unit cost in all cases. ^(vi)^ In the CAPI at Wave 1 and 2, these frequencies were binary ( *i.e.*, do you use this service?); for non-users we set *y _i,h_* =0; for those using the service we set *y _i,h_* to equal the age- and sex-adjusted median among service users in Waves 3-5. ^(vii)^ Brick *et al.*, estimated costs for 2011; per the CSO CPI Health, the multiplier from December 2011 to December 2022 was 1.122. ^(viii)^ ‘Home help’ in TILDA is termed ‘Health Care Support Assistant’ in Smith *et al.* ^(ix)^ For ‘Home care package’ and ‘Personal care attendant’ in TILDA, we used ‘Health Care Support Assistant’ in Smith *et al.* ^(x)^ For ‘Nursing home’ in TILDA, we used ‘Long-term residential care’ in Smith *et al.* ^(xi)^ Hospice use is not part of the CAPI. CAPI, computer-assisted personal interview; EOLI, end-of-life interview; CSO, Central Statistics Office; CPI, Consumer Price Index; HIPE, Hospital Inpatient Enquiry; TILDA, The Irish Longitudinal Study on Ageing.

For medications, CAPI respondents detail the medications that they take “on a regular basis”, which includes prescribed medications, as well as those purchased over-the-counter, vitamins and supplements, and herbal products. Medication names are recorded as they are reported (either branded/generic product name or drug name), however strength and dosage are not captured. Each medication is assigned a WHO Anatomical Therapeutic Chemical (ATC) code where available relating to the drug they contain. We excluded reported products that do not have an ATC code, and any non-prescription items not reimbursed on Ireland’s community drug schemes ( *i.e.*, certain vitamins and over-the-counter products). For each included medication ( *m*) we identified the associated cost ( *c*), assuming the respondent was prescribed the WHO Defined Daily Dosage corresponding to the ATC code for one year, in the 2020 Health Service Executive reimbursement list ^46^. Therefore, for each individual CAPI ( *i*), reported regular usage of *n* medications has associated annual costs ( *y _i,m_*) given by:


$$y_{i,m}=\sum_{m=1}^{n}c_{i,m}$$

The EOLI does not collect medications data, but age- and sex-adjusted mean costs in the last year of life have been calculated previously ^47^. We imputed into EOLIs *y _i,m_* using this mean by age and sex, after adjusting to 2022 using the CPI for health.

The primary outcome, an individual CAPI or EOLI’s total formal health care costs ( *Y _i_*), expressed in euro (€, EUR) adjusted to 2022, is then calculated by summing *y _i,h_* for *n* categories of acute and non-acute care and adding the medications costs:


$$Y_{i}=\sum_{h=1}^{n}y_{i,h}+y_{i,m}$$

This outcome variable does not include some CAPI formal health care use data that might be considered relevant to health care costs. These variables, and the rationale for not including in this paper, are summarised in Table 2.

**Table 2.** Formal health care utilisation categories recorded in TILDA but excluded from this paper.

Category	CAPI ^48^	Reason for exclusion
Optician	hu015_12	No unit cost reported in Brick *et al.*, Smith *et al.*, or PSSRU 2019 ^49^
Hearing	hu015_14	
Respite care	hu015_16	
Consultant	hu062	Binary, no frequency data to calculate costs
Operations	hu011	No operation-specific data on which to base unit costs.
Public or private hospital?	hu014	No unit costs available for private hospitals.
Private home care	hu076	Not collected for all CAPI waves, and/or in all EOLI waves; excluded for consistency.
Private allied health and social care	hu084	

CAPI, computer-assisted personal interview; EOLI, end-of-life interview; TILDA, The Irish Longitudinal Study on Ageing.

 We did not identify any health care use variables in the EOLI that are not in either Table 1 or Table 2. Those interested may check the full suite of TILDA variables at any time *via* the study website.

***Independent variables***

In multivariate regressions for our third research question, we identified predictors on a hypothesis-driven basis using the Andersen model of health care utilisation, which categorises potential predictors as predisposing, enabling, need or prior use ^50^. Additionally we controlled for proximity-to-death effects using death dates for both CAPI and EOLI observations. The variables employed in multivariate regressions are summarised in Table 3.

**Table 3.** Independent predictors in multivariate regression.

Group	Variable	Categorisation
***Predisposing***	Age ^§^	Years
	Sex ^§^	Male | Female
***Enabling***	Education: Highest achieved ^§^	Primary | Secondary | Tertiary
	Medical card or GP card? * ^#^	Yes, either/or
	Private insurance? * ^Ɛ^	Yes, either as policy holder or on another’s policy
	Marital status	Married | Living with a partner ==1 Single | Widowed | Divorced | Separated ==0
	Local region *	Dublin city and county | Urban area, not Dublin | Rural area
***Need***	Cancer ^¥^	Has a doctor ever told you that you have ___?
	Heart disease ^¥^	Has a doctor ever told you that you have ___?
	Multimorbidity ^¥^	Has a doctor ever told you that you have 2+ of the following: cancer, heart disease, kidney disease, liver disease, lung disease, Alzheimer’s disease and related dementias, hypertension; diabetes; stroke; arthritis; psychological issues including anxiety and depression; alcohol and/or drug abuse?
	Instrumental Activities of Daily Living (IADLs) * ^51^	Because of a health or memory problem, do you have difficulty doing any of the following activities: preparing a hot meal, shopping for groceries, making telephone calls, taking medications, managing money, doing household chores? Total difficulties (/6)= 0 | 1 | 2+
	Physical exercise ^β^	Do you engage in vigorous physical exercise at least weekly?
***Prior use***	Emergency department (ED) admissions ^+^	How many ED admissions in the prior interview? Total admissions = 0 | 1 | 2+
***Proximity to*** ***death***	Last two years of life (L2YOL) ^θ^	Among CAPI sample, did the participant die within two years of the interview?
	Last year of life (LYOL) ^α^	Was the participant in the last year of life ( *i.e.*, is this a CAPI observation or an EOLI observation)?

^§^ For both CAPI and EOLI observations, these variables are taken from the baseline enrolment data (and EOLI age adjusted to age at death using date of death). ^#^ Medical cards are provided on a means-tested basis, and provide free access to inpatient and outpatient public hospital care, to GPs, and to prescription drugs; GP cards are means-tested under the age of 70 years and provided universally thereafter, and afford free access to the GP. ^52^ ^Ɛ^ Private insurance is voluntary in Ireland; it provides access to some additional facilities and expedites access to certain services. * For CAPI observations, these variables are taken from the Wave 5 responses: for EOLI observations, these variables are taken from the EOLI or if the respondent did not know or refused to answer they are taken from the last CAPI prior to death. ^¥^ Diagnoses are treated as absorbing states; for CAPI observations, a reported diagnosis at any Wave up to and including Wave 5; for EOLI observations, a reported diagnosis at any CAPI or in the EOLI. ^β^ For CAPI observations, these variables are taken from Wave 5; for EOLI observations, these variables are taken from the last CAPI prior to death. ^+^ For CAPI observations, prior use variables are taken from Wave 4; for EOLI observations, these variables are taken from the last CAPI prior to death. ^θ^ Identified *via* GRO linkage; included in CAPI and pooled regressions only; always ==0 in EOLI sample. ^α ^Included in pooled regression only, has a fixed value within CAPI (==0) and EOLI (==1) samples. CAPI, computer-assisted personal interview; EOLI, end-of-life interview; GRO, General Register Office.

### Sample eligibility and timeframe for analysis

In the main paper we focus on two sets of interviews: CAPIs at Wave 5, and EOLIs at any wave. We choose Wave 5 as the most recent conducted prior to the COVID-19 pandemic; Wave 6 interviews were conducted during 2021, which was an atypical period of health care utilisation and is likely not generalizable to other years. By Wave 5 (2018), the baseline sample (aged 50+ years in 2009–2011) are nearly all aged 55+ years (the only exceptions are those who enrolled aged <50 years old while participating as the spouse of a participant aged >50 years old). Therefore, we excluded those aged <55 years from all analyses; the numbers are presented with the population-level weights in ‘Appendix 2’, found as *Extended data* ^42^.

We include EOLIs from all waves prior to March 2020, since wave-by-wave samples are relatively small, these observations heavily influence cost estimates (see Results for full details), and we consider it a reasonable assumption that pre-pandemic deaths in all TILDA years are substantively comparable. The sample eligibility was therefore defined as all Wave 5 CAPI participants aged 55+ years, and all EOLIs aged 55+ years at any wave, except (i) deaths occurring after 29/2/20, and (ii) the deaths for participants at Wave 5 and so individuals already in our sample. We summarise how this sample is reached in the Results and present the characteristics of those excluded in ‘Appendix 2’ ^42^.

As such our reported estimates reflect our best understanding of health care costs among older people in Ireland in 2019, updated for inflation to 2022.

### Missing data, final sample size and sensitivity analyses

Prior studies have found that missing data in both CAPIs and EOLIs is relatively rare; *e.g.*, at baseline this was less than 1% for predisposing, enabling and need characteristics ( Table 3 in May *et al.*, 2022 ^33^), although there have been small increases in such missingness wave-on-wave. For the dependent variable, prior analyses of TILDA have suggested that of all categories in Table 1, four account for over 80% of total costs in the CAPI: GP, inpatient, outpatient and home help ^27, 28^. Any sample-eligible CAPI or EOLI that was missing two or more of these four categories was flagged and removed from primary analysis as having insufficient outcome data. For those interviews missing one or fewer of these categories, and or missing any other categories of health care frequency, we imputed age- and sex-adjusted medians. For independent variables, any sample-eligible CAPI or EOLI that was missing three or more baseline predictors was removed from primary analysis as having insufficient baseline data. For those interviews missing two or fewer baseline variables, we imputed the same individual’s data from the most recently available prior wave.

### Bias

TILDA in Wave 1 aimed to recruit a population-representative sample of community-dwelling adults aged 50+ years but the sample inevitably differs from the population, and this variation will have increased if those who die or drop out or have missing data differ systematically from those who continue to take part. We addressed this sampling uncertainty, and the concomitant risk of bias, through sampling weights that used the CSO population data to calculate the probability of any given participant having been included in the sample. For this paper we weighted by age (five-year bands), sex (male or female), and last year of life (=1 for EOLIs, 0 for CAPIs). Weights were calculated using the CSO population data for 2019, the most recent pre-pandemic year full data were available. See ‘Appendix 2’ ^42^.

### Statistical methods

All analyses were performed in Stata version 15 (RRID:SCR_012763) ^53^; an open access alternative that can perform equivalent tasks is R (RRID:SCR_001905) ^54^. For research questions 1 and 2, we report descriptive and distributional statistics after applying the population weights. For research question 3, we run multivariate regressions in the eligible Wave 5 CAPIs, in the eligible EOLIs, and in the CAPIs and the EOLIs pooled. In all cases we modelled outcomes using a generalised linear model with a power link, selected using information criteria before inspecting or interpreting results ^55^. Prior to estimating results we assessed collinearity of predictors using the –collin- command. For each association between predictor and outcome, we report dy/dx using the –margins- command; this reflects the estimated mean association with outcome of increasing the value of the predictor by one point while holding all other values in the model constant.

***Additional data and sensitivity analyses***

**Figure 1.**
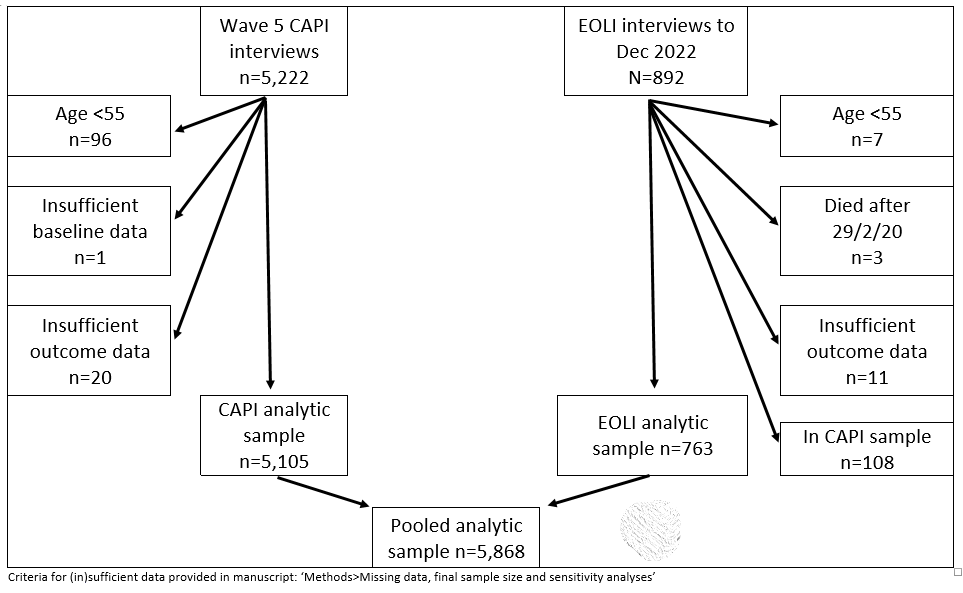
How the analytic samples were reached. CAPI, computer-assisted personal interview; EOLI, end-of-life interview.

For reader information, we present the following data before and after weighting in supplementary materials:

• Characteristics of the sample, and those excluded per Figure 1 (Appendix 2) ^42^
• Research Question 1 (Appendix 3, which can be found as *Extended data*) ^42^
• Research Question 2 (Appendix 4) ^42^
• Summary statistics and distributions for total health care costs in CAPI waves 1–4 (Appendix 5) ^42^

We performed sensitivity analyses on our regressions, presented in Appendices:

I. Research Question 3 for CAPI and EOLI without weights (Appendix 6) ^42^
II. Research Question 3 for CAPI and EOLI with alternative acute inpatient costs as outlined in Appendix 1 (Appendix 6) ^42^
III. Research Question 3 for CAPI and EOLI using those with complete outcome data only (Appendix 6) ^42^

Diagnostic checks for model choice and collinearity are presented in ‘Appendix 7’ ^42^. Additional information on the medications costing exercise are presented in ‘Appendix 8’ ^42^.

## Results

### Sample

Figure 1 details how the analytic samples were reached. There were 5,222 completed CAPI interviews at Wave 5, of which 96 were aged <55 years, one had insufficient baseline data, and 20 had insufficient outcome data. This gave a CAPI analytic sample of 5,105 Wave 5 participants. There were 892 completed EOLI interviews at time of data analysis (Q3 2022), of which seven were for people aged <55 years, three concerned deaths occurring March 2020 onwards, 11 had insufficient outcome data, and 108 were already in the CAPI sample. This gave an EOLI analytic sample of 763 deceased participants. When the CAPI and EOLI data were pooled, this was an analytic sample of 5,868 unique individuals ( Figure 1).

### Descriptive data

The analytic samples are provided in Table 4, after weighting. The 5,105 CAPI observations were equivalent to 1,207,660 people at the population level, and the 763 EOLI observations were equivalent to 28,466. Combined this was 1,236,126 people aged 55+ years in Ireland in 2019.

**Table 4.** Baseline characteristics, all samples, after weighting.

Baseline characteristics		CAPI (N _TILDA_=5,105)		EOLI (N _TILDA_ =763)		ALL (N _TILDA_ =5,868)	
		N _pop_=1,207,660		N _pop_=28,466		N _pop_=1,236,126	
		%	n _pop_	%	n _pop_	%	n _pop_
**Age**	*55–64yrs*	44.4%	536,486	10.2%	2,903	43.6%	539,389
	*65–74yrs*	33.0%	398,755	19.8%	5,629	32.7%	404,384
	*75–84yrs*	17.1%	205,879	31.4%	8,942	17.4%	214,821
	*85yrs<=*	5.5%	66,210	38.6%	10,992	6.2%	77,202
**Sex**	*Male*	48.0%	579,195	51.1%	14,545	48.0%	593,740
**Education**	*Primary*	19.9%	240,498	53.1%	15,105	20.7%	255,603
	*Secondary*	43.3%	522,604	29.4%	8,374	42.9%	530,978
	*Tertiary*	36.8%	444,558	17.5%	4,987	36.4%	449,545
**Married**	*Yes*	70.3%	848,914	41.8%	11,890	69.7%	860,804
**Medical card**	*Yes*	55.0%	663,481	91.5%	26,055	55.8%	689,536
**Insurance**	*Yes*	61.9%	747,063	37.9%	10,801	61.3%	757,864
**Region**	*Dublin*	24.3%	293,099	21.6%	6,166	24.2%	299,265
	*Urban, not Dublin*	29.0%	350,345	32.4%	9,211	29.1%	359,556
	*Rural area*	46.7%	564,216	46.0%	13,089	46.7%	577,305
**Diagnoses**	*Cancer*	10.6%	127,571	42.4%	12,057	11.3%	139,628
	*Heart disease*	27.3%	329,769	47.5%	13,518	27.8%	343,287
	*Multimorbidity*	41.4%	499,818	82.4%	23,443	42.3%	523,261
**IADLs**	*0*	92.6%	1,118,607	35.8%	10,192	91.3%	1,128,799
	*1*	2.6%	30,933	20.9%	5,955	3.0%	36,888
	*2+*	4.8%	58,120	43.3%	12,319	5.7%	70,439
**Phys. exercise**	*Yes*	24.9%	300,950	5.5%	1,562	24.5%	302,512
**ED visits**	*0*	84.7%	1,023,048	67.9%	19,340	84.4%	1,042,388
	*1*	12.3%	148,229	19.0%	5,403	12.4%	153,632
	*2+*	3.0%	36,383	13.1%	3,723	3.2%	40,106
**L2YOL**	*Yes*	2.1%	21,584	-	-	1.8%	21,584
**LYOL**	*Yes*	-	-	100%	28,466	2.3%	28,466

N _TILDA_ is the number of observations in each sample (CAPI/EOLI/ALL). N _pop_ is the number of people in each sample (CAPI/EOLI/ALL) at the population level after weighting; n _pop_ is the number of people in each cell at the population level after weighting. For unweighted samples and TILDA cell sizes, see ‘Appendix 2’ in *Extended data* ^42^. For definitions of variables, see Table 3. CAPI, computer-assisted personal interview; EOLI, end-of-life interview; TILDA, The Irish Longitudinal Study on Ageing; IADL, Instrumental Activities of Daily Living; ED, Emergency department; L2YOL, last two years of life; LYOL, last year of life.

There were large differences between the EOLI (last year of life) and CAPI (not last year of life) samples on all baseline predictors except region. More than two thirds of those in the last year of life were aged over 75 years, and more than three quarters not in the last year of life were aged under 75 years. Males were slightly more represented among the EOLI than CAPI samples, reflecting higher male mortality rates. The younger CAPI interviewees had higher average educational achievement, reflecting cohort effects in access, and higher prevalence of marriage, reflecting rising marriage rates in the middle of the 20 ^th^ century and lower widowhood effects. Those in the last year of life were much more likely to have a medical card, reflecting wider entitlement from the age of 70 years, but less likely to have private insurance. EOLI observations had much higher prevalence of serious illness, functional impairment, and ED attendance; but much lower prevalence of regular physical exercise. In the pooled sample, patterns of characteristics substantively reflect the CAPI sample as these are 87% of observations before weighting and 98% after weighting.

### Main results

***What are the health care costs for older people in Ireland? How are costs distributed across the population?***

Total formal care costs, in the CAPI and EOLI samples and pooled together, are presented in Table 5. Mean costs in the weighted sample were EUR 8,053, comprising EUR 6,624 in the CAPI sample and EUR 68,654 in the EOLI. Typical for cost data, there is considerable right-hand skew in all samples.

**Table 5.** Distribution of estimated mean formal costs (2022 EUR), after weighting.

*Mean*	CAPI	EOLI	ALL
	EUR 6,624	EUR 68,654	EUR 8,053
***Percentile***			
*10th*	EUR 98	EUR 12,806	EUR 98
*25th*	EUR 328	EUR 28,077	EUR 332
*50th*	EUR 848	EUR 49,974	EUR 887
*75th*	EUR 2,545	EUR 95,326	EUR 2,945
*90th*	EUR 16,500	EUR 131,865	EUR 18,775
*Largest*	EUR 671,529	EUR 945,983	EUR 945,983

CAPI, computer-assisted personal interview; EOLI, end-of-life interview.

**Figure 2.**
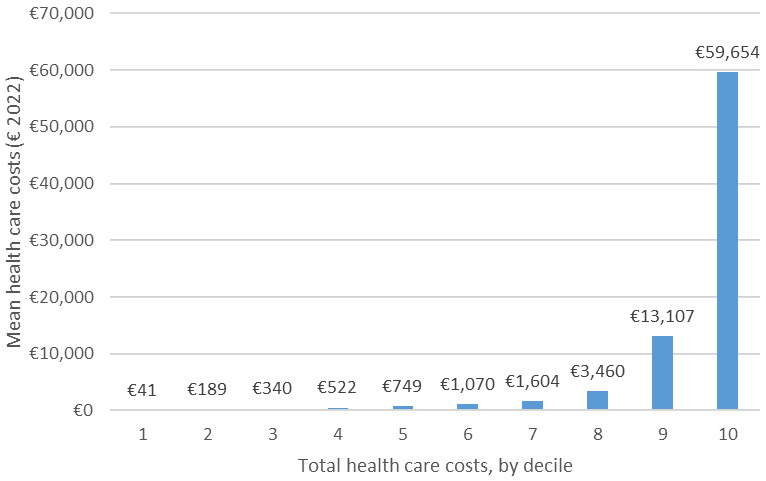
Distribution of costs by decile in pooled (ALL) sample.

The distribution of formal costs across deciles are presented in Figure 2, after weighting. The skew is again heavily evidenced here: over 70% of people have costs less than EUR 2,000 a year, and the top 10% of people have mean costs of EUR 59,654.

The corresponding population-level costs are presented in Table 6. Total estimated population-level costs are EUR 9,954,054,582. Almost three quarters (74%) of these costs are accounted for by the 10th decile and another 16% by the ninth decile. Therefore, an estimated 90% of health care costs among people aged 55+ years are accounted for by 20% of users.

**Table 6.** Distribution of population-level costs by decile in pooled (ALL) sample.

Decile	Total costs	% of TOTAL
1	EUR 6,124,598	<0.5%
2	EUR 18,333,207	<0.5%
3	EUR 42,035,469	<0.5%
4	EUR 64,543,771	1%
5	EUR 92,453,829	1%
6	EUR 132,231,904	1%
7	EUR 198,341,926	2%
8	EUR 427,823,171	4%
9	EUR 1,624,557,991	16%
10	EUR 7,347,608,716	74%
*TOTAL*	EUR 9,954,054,582	

 For equivalent data in the CAPI and EOLI samples separately, see Appendix 3 ^42^.

***What is the underlying composition of these costs between primary and community care, hospital care, home care, residential care, and medications?***

**Figure 3.**
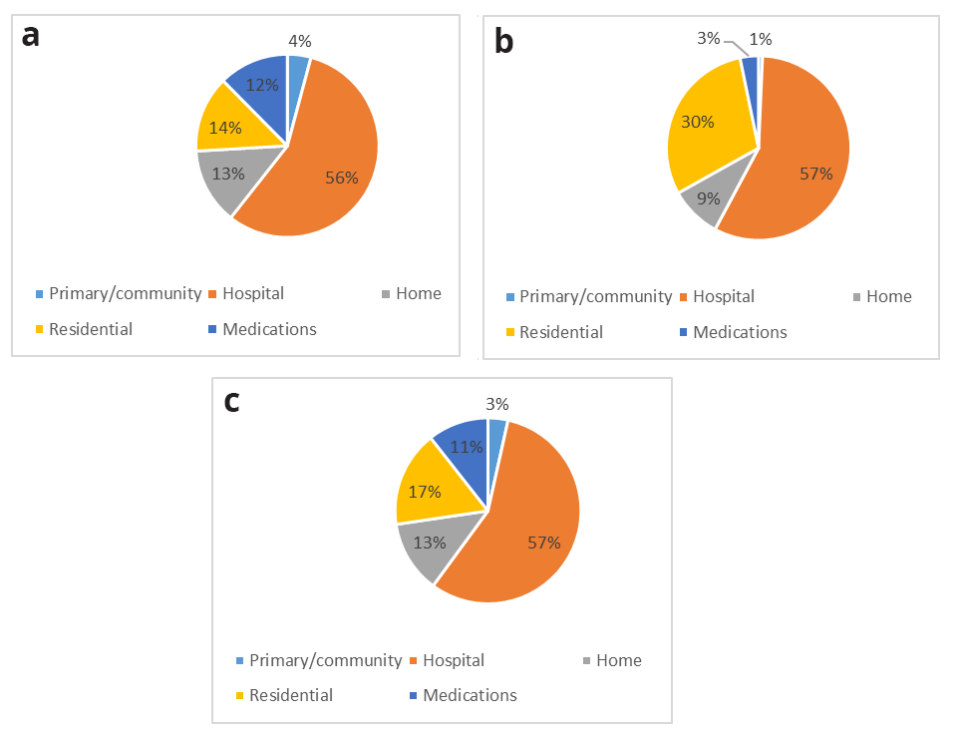
Composition of costs. ( **a**) CAPI, ( **b**) EOLI and ( **c**) pooled (ALL) sample. CAPI, computer-assisted personal interview; EOLI, end-of-life interview.

The composition of costs for CAPI, EOLI and pooled samples are presented in Figures 3a, 3b and 3c, respectively. Hospital costs accounted for the majority of the dependent variable in all three cases; the most visible difference between CAPI and EOLI costs were those for residential care, which accounted for a far higher overall proportion among those in the last year of life.

**Figure 4.**
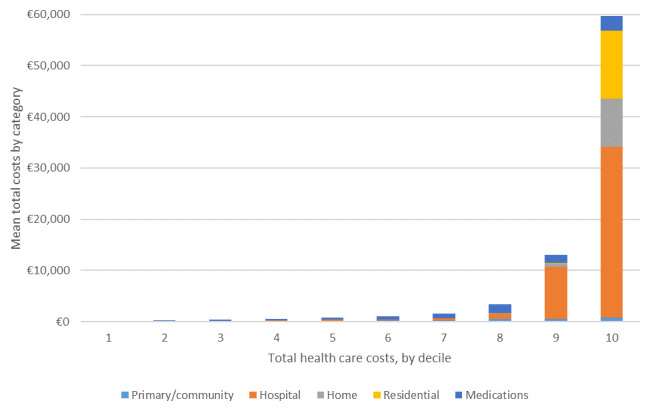
Distribution and composition of costs by decile in pooled (ALL) sample.

The composition of costs across deciles of total health care costs in the pooled sample is presented in Figure 4. The substantive jump between the eighth and ninth decile, already highlighted in Table 6, is mainly explained by hospital costs. The even larger jump between the ninth and 10th decile is explained by large increases in hospital, home and residential care costs.

**Figure 5.**
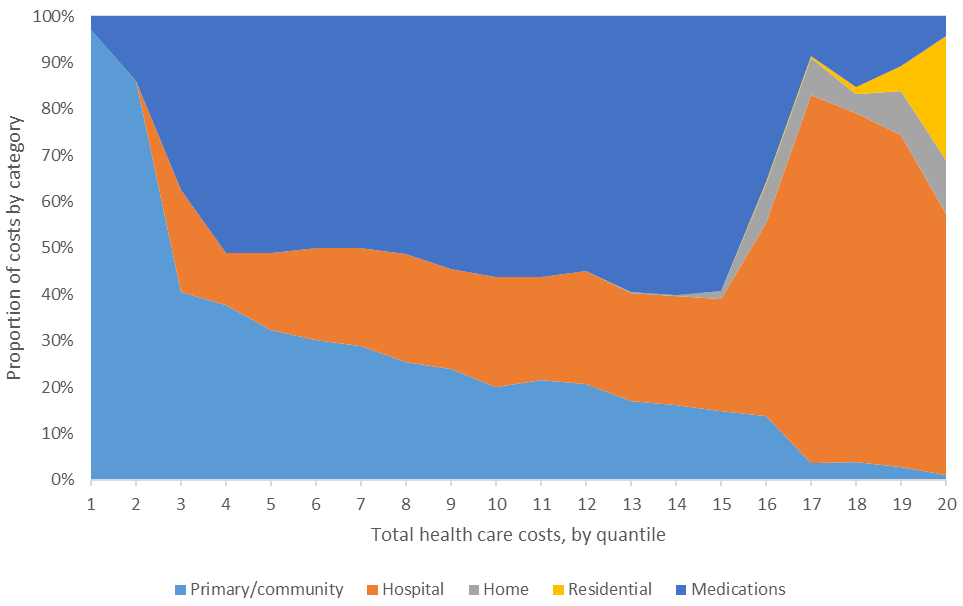
Composition of costs by quantile in pooled (ALL) sample.

The composition of costs among non-zero users, across 20 quantiles of total health care costs in the pooled sample, is presented in Figure 5. Among lower cost users, primary care and pharmacy costs dominate. From the second to 17 ^th^ quantile, hospital costs account for a consistently increasing proportion. Home care costs are close to zero until the top five quantiles, thereafter, accounting for 2–12%. Residential care costs are close to zero until the top two quantiles, accounting for over a quarter of costs in the highest-cost group.

 For equivalent data in the CAPI and EOLI samples separately, see Appendix 4 ^42^.

***What individual-level predictors are associated health care costs?***

The results of the multivariate regressions are presented in Table 7. Statistically significant results are highlighted in bold.

**Table 7.** Associations between individual characteristics and total health care costs.

Individual characteristics		CAPI		EOLI		ALL	
		*dy/dx*	*95% CI*	*dy/dx*	*95% CI*	*dy/dx*	*95% CI*
**Age**	*65-74yrs*	**699**	**118 to 1,279**	13,401	-1,151 to 27,954	**747**	**170 to 1,324**
	*75-84yrs*	765	-320 to 1,850	3,053	-11,299 to 17,406	818	-261 to 1,897
	*85yrs<=*	**3,639**	**1,132 to 6,147**	-35	-14,658 to 14,587	**3,776**	**1,337 to 6,215**
**Sex**	*Male*	-242	-704 to 219	**-11,764**	**-20,624 to -2,904**	-260	-719 to 198
**Education**	*Primary*	-224	-1,025 to 576	-653	-10,710 to 9,404	-241	-1,036 to 554
	*Tertiary*	**-768**	**-1,295 to -241**	3,794	-9,053 to 16,642	**-817**	**-1,341 to -294**
**Married**	*Yes*	-256	-871 to 359	-110	-9,387 to 9,167	-273	-884 to 339
**Med. card**	*Yes*	**1,346**	**665 to 2,028**	6,805	-8,512 to 22,122	**1,434**	**757 to 2,112**
**Insurance**	*Yes*	491	-78 to 1,060	-2,695	-13,240 to 7,849	520	-46 to 1,086
**Region**	*Urban*	**1,048**	**316 to 1,780**	**-16,025**	**-29,642 to -2,408**	**1,109**	**383 to 1,835**
	*Rural*	-384	-948 to 180	**-17,840**	**-30,812 to -4,869**	-413	-975 to 149
**Diagnoses**	*Cancer*	**3,890**	**2,361 to 5,419**	**14,796**	**5,810 to 23,782**	**4,166**	**2,647 to 5,685**
	*Heart*	**2,185**	**1,299 to 3,071**	6,444	-3,075 to 15,963	**2,328**	**1,448 to 3,208**
	*Multim.*	**2,679**	**1,930 to 3,428**	**17,580**	**6,964 to 28,196**	**2,855**	**2,111 to 3,598**
**IADLs**	*1*	1,969	-723 to 4,660	**11,572**	**799 to 22,345**	2,147	-517 to 4,811
	*2+*	**21,376**	**11,714 to 31,037**	**30,637**	**20,287 to 40,987**	**21,437**	**12,763 to 30,112**
**Exercise**	*Yes*	**-1,645**	**-2,137 to -1,154**	**-24,751**	**-39,619 to -9,884**	**-1,751**	**-2,240 to -1,263**
**ED visits**	*1*	**1,737**	**663 to 2,812**	6,968	-4,853 to 18,789	**1,844**	**784 to 2,905**
	*2+*	**4,542**	**915 to 8,170**	**15,717**	**67 to 31,366**	**4,837**	**1,282 to 8,392**
**L2YOL**	*Yes*	**16,250**	**7,304 to 25,195**	-	-	**17,325**	**8,439 to 26,210**
**LYOL**	*Yes*	-	-	-	-	**17,865**	**9,875 to 25,855**

For variable definitions, including reference cases, see Table 3. For full regression output including p-values, see Appendix 6 in *Extended data* ^42^. dy/dx: the marginal effect; the estimated mean association with outcome of increasing the value of the predictor by one point while holding all other values in the model constant. CI, confidence interval; CAPI, computer-assisted personal interview; EOLI, end-of-life interview; IADL, Instrumental Activities of Daily Living; ED, Emergency department; L2YOL, last two years of life; LYOL, last year of life.

In the CAPI sample, the largest associations were 2+ IADLs, which was associated with EUR 21,376 higher costs compared to none (95% confidence interval: 11,714 to 31,037) and being in the last two years of life (+ EUR 16,250; 7,304 to 25,195). Other variables positively associated with outcome were: older age; medical card entitlement; living in a town or city other than Dublin; diagnoses of cancer, heart disease or multimorbidity; and prior ED attendance. Negative associations were college education and engaging in regular physical exercise, which in this context is a proxy for unobserved general health. In the EOLI sample, the largest association was again 2+ IADLs (+ EUR 30,637; 20,287 to 40,987). Other positive associations were cancer diagnosis and multimorbidity. Negative associations were male sex, living outside Dublin, regular exercise and multiple prior ED attendances.

In the pooled sample, results were substantively consistent with the CAPI, reflecting the fact that these are 98% of observations in the weighted sample. The largest associations were again multiple IADLs (+ EUR 21,437; 12,763 to 30,112), and proximity to death variables in the last two years of life (+ EUR 17,325; 8,439 to 26,210) and in the last year of life (+ EUR 17,865; 9,875 to 25,855).

## Discussion

### Key findings

This paper presents the most comprehensive picture to date on individual-level health care costs for older people in Ireland. We found that, adjusted to end 2022, mean costs among people aged 55+ years were EUR 8,053, with a large differential between those in the last year of life (EUR 68,654), and not in the last year of life (EUR 6,624). Hospital costs accounted for over half of costs in all three sampling frames. There was a very large skew in the data: the top 20% of users accounted for 90% of all costs at the population level, and the top 10% accounted for 74%. In multivariate regressions, multiple IADLs and proximity to death had the largest associations with outcome. In sensitivity analyses, our results are substantively similar for the IADL and proximity to death associations, and >95% of all associations in Table 7 have the same interpretation in these sensitivity analyses (Appendix 6) ^42^.

### Interpretation

The large association between multiple IADLs and costs is not surprising, and in part reflects the high level of health care worker time that are required to support this population. Nevertheless, the magnitude of association, and in particular that this variable is more strongly predictive of costs than proximity to death, is somewhat unexpected and has important policy implications. As the population ages, and in particular as dementia becomes more prevalent, the number of people living with multiple IADLs will grow ^33, 56– 58^. Optimising care and supports for this group, in particular to support ageing in place and to minimise avoidable acute hospital admissions and residential care costs, remains an urgent priority ^29– 32^.

The significant differential between those in the last two years of life and not approaching end of life reflects a long-standing evidence base that proximity to death is a key determinant of health care costs. There were 31,184 deaths recorded in Ireland in 2019, of which over 28,000 (91%) occurred in people aged over 55 years ^59^. The population-level costs of end-of-life care are therefore approximately EUR 2 billion annually. Total health spending in Ireland in 2019 was EUR 25.3 billion ^60, 61^. This suggests that the <1% of people who die each year in Ireland account for around 8% of health care spending, although the true ratio is sensitive to recent rapid increases in both inflation and public health spending, and the fact that our estimates likely undervalue total spend by using public service unit costs only. This ratio is consistent with what has been reported in other high-income countries ^62^. The large projected growth in the number of older people dying annually as the population ages emphasises the urgent need to plan and fund palliative and end-of-life care services.

Some of the other associations were predictable in the context of prior literature. Older age is associated with somewhat higher costs compared to younger people (though this is heavily tempered by proximity-to-death dynamics, and more observable for social care than health care) ^5^. Men have lower costs in the last-year-of-life cohort due to the higher prevalence of sudden death, but there is no apparent association in the whole population. Socioeconomic disparities in health are reflected in lower costs among those who stayed longer in education ^63^. Medical card entitlements lead to higher health care use in the general population ^64, 65^, but in the end-of-life cohort where entitlements are more universal, they have no relationship. This relationship between entitlements and costs is complicated by potential confounding by socioeconomic and health status; those entitled to a medical card are more likely to have care needs that are not captured in the model. Diagnoses of serious disease necessitate higher health care costs ^66– 68^ and regular physical exercise protects against episodes of ill-health ^69, 70^, although in our data this is likely not causal but reflects the better health of regular exercisers. Prior patterns of hospital admittance strongly predict costs ^71^. One surprising result was the association between region and outcome: those living outside Dublin in other urban settings had higher costs in the general population, but both urban and rural dwellers had substantially lower costs than those in the capital in the end-of-life cohort. Complex patterns of use by geographical region are commonplace among older populations ^72, 73^, and this warrants further investigation to pick apart issues of need, access and value ^74^.

While skew in health care costs is a long-established phenomenon, the distribution in our data is still unusual. The historic interest has been in an ‘80–20 ratio’, where 20% of people account for 80% of spending, but we find that 20% of people account for 90%. Taking into account that our calculations are in the population aged 55+ years, and older people account disproportionately for health care spending, the ratio at the population level must be still more imbalanced. Ex ante identification of people who account heavily for health care costs, identifying and addressing low-value care, and reforming provision for an age of multimorbidity and ongoing supportive care are all strategies with enormous potential fiscal pay-offs, as well as improved outcomes for patients.

The estimation that hospital costs account for nearly 60% of total costs is higher than comparable data in England (50%) ^75^. This is potentially associated with Ireland’s historic reliance on acute care and weak primary care capacity, and implies opportunity to reduce hospital costs through more cost-effective models of community care delivery ^16, 21^. Such aims are consistent with the ongoing Sláintecare reforms.

### Limitations

TILDA collects all CAPI data by self-report, and all EOLI by interview with a family member or friend, which may result in omissions and recall bias among both predictors and outcome. Absent a unique identifier in routine data, TILDA is nevertheless among the most powerful sources of individual-level data for understanding health care costs among older people in Ireland.

Our unit cost estimates do not take account of differential costs in private settings, which by volume account for approximately 20% of care in Ireland’s mixed system ^18, 65^. This means that our total reported costs are likely underestimates, and that interpreting associations between outcome and variables strongly associated with use of private care ( *e.g.*, health insurance, socioeconomic status) must be done with caution. TILDA collects frequency data on private hospital and home care use, meaning that future work can address this gap if credible unit costs of private care can be identified. Proportion of use that is private among different allied and social care categories varies widely, and as such so does the public/private distinction. We are unable to quantify the distribution of costs between payers (public and private care, and out-of-pocket costs). Many hospital engagements in Ireland are ‘day cases’; engagements not requiring an overnight stay. While TILDA captures ED attendances and same-day discharge following emergency admissions are included in our reported costs, we don’t include diagnosis-specific costs for outpatient engagements or procedures but instead estimate resources at a flat unit cost rate. Healthcare Pricing Office costs for inpatient stays do not include superannuation, while unit costs for other types of care do.

Hospital costs account for a majority of overall costs, but unit costs for acute care are age/sex/diagnosis-adjusted national averages only. While we have adjusted for discharge status (alive/dead; see Appendix 1) ^42^, and age/sex/diagnostic profiles capture a significant proportion of proximity to death among those discharged alive, hospital costs are still highly heterogeneous within each age/sex/diagnostic category, reflecting myriad factors including physician and patient preferences, access, specific hospital setting, and discharge location options. Future work might address this to some extent; *e.g.*, HIPE records whether a person was discharged home or to a hospice or a nursing home. However, arising estimated costs would be contingent on additional unverifiable assumptions and so come with increased risk of new biases. The promised implementation of a unique identifier would provide ‘true’ individual-level costs in HIPE against which different cost mix methods in TILDA could then be benchmarked.

The COVID-19 pandemic complicated choice of an appropriate timeframe. For CAPI observations, we used Wave 5 as the most recent pre-pandemic wave (2018), and for EOLI observations, we used all pre-pandemic observations to maximise sample size in a group that has disproportionate influence on estimates. Our reported estimates reflect best understanding of health care costs among older people in Ireland in 2019, updated for inflation to 2022. The information provided in this manuscript and in the appendices equips readers to revise group averages using the CPI for health, and/or to weight at the population level for other years using CSO data, should they choose to do so. Parsing the effects of COVID-19 on general health care use, both during the heights of the pandemic 2020–2021, and into the future, have been examined to some extent in other TILDA analyses and are an important topic for ongoing study.

TILDA recruited a population-representative sample in 2009–2011, but attrition to Wave 5 (2018) may have undermined this representativeness. We weighted using age, sex and last year of life since these data are theoretically associated with outcome and easily available from the CSO. Prior weighting exercises in the TILDA CAPI have also incorporated education, marital status and geographical location to maximise generalisability ^76^. While these data are available *via* the census for the CAPI, the last matching exercise by the CSO to the GRO was after the 2016 Census. Our strategy therefore reflects the best approach with publicly available data for all CAPI and EOLI observations. Future work may seek to improve the precision of weighting, for example by getting additional ‘enabling’ variables on decedents from the CSO’s data controllers.

## Conclusions

High-quality data on health care costs are essential to support monitoring, planning and evaluation of services, and the allocation of scarce resources to maximise public welfare. By combining newly available unit cost data in non-acute care, our own estimates of acute costs, and the rich data in TILDA, we present the most comprehensive picture to date on individual-level costs among older people in Ireland. We quantify more precisely some well-known relationships, particularly the high costs associated with end-of-life care, and also identify some potentially underestimated dynamics, in particular that multiple functional impairments appear a more significant driver of costs than age, diagnosis, multimorbidity or proximity to death. The derived estimates can inform multiple ongoing research studies and policy activities, as well as providing a foundation for future work, which should include consideration of private provider costs in Ireland’s unusual mixed system.

## Consent

Ethical approval for each wave of the TILDA study is obtained from the Faculty of Health Sciences Research Ethics Committee in Trinity College Dublin. Participants are provided with sufficient information to make an informed decision about their participation including advance notice of the study. Written consent is obtained for separate components of the study (i.e., interview, health assessment, blood samples); participants may refuse to take part in or withdraw at any time without justification. Ethical approval for the secondary analysis of TILDA data used in this study was part of this overall approval.

## Data Availability

Researchers interested in using regular waves of TILDA data may access the data for free from the following sites: Irish Social Science Data Archive (ISSDA) at University College Dublin ( http://www.ucd.ie/issda/data/tilda/); Interuniversity Consortium for Political and Social Research (ICPSR) at the University of Michigan ( http://www.icpsr.umich.edu/icpsrweb/NACDA/studies/34315). Replication of the results reported in this article requires access to the full TILDA dataset, which is held on secure servers at the study site at Trinity College Dublin (TCD). Researchers seeking access to the full TILDA dataset may apply to access the data on the TCD campus ( tilda.tcd.ie); applications are considered on a case-by-case basis; all Stata do files and code employed in this paper will be made available to applicants on request. Open Science Framework: Appendices to 'Formal health care costs among older people in Ireland: methods and estimates using The Irish Longitudinal Study on Ageing (TILDA)'. https://doi.org/10.17605/OSF.IO/76SYK ^42^. This project contains the following extended data: Appendix 1.docx (information on calculations of costs associated with inpatient hospital admissions in TILDA) Appendix 2.xlsx (characteristics of the sample, and those excluded per Figure 1) Appendix 3.docx (calculations of health care costs for older people in Ireland and distribution across the population) Appendix 4.docx (calculations of underlying composition of health care costs between primary and community care, hospital care, home care, residential care, and medications) Appendix 5.docx (summary statistics and distributions for total health care costs in CAPI waves 1–4) Appendix 6.docx (sensitivity analysis) Appendix 7.docx (diagnostic checks for model choice and collinearity) Appendix 8.docx (information on the medications costing exercise) STROBE checklist Data are available under the terms of the Creative Commons Attribution 4.0 International license (CC-BY 4.0).

## References

[1] Organisation for Economic Co-operation and Development: Health at a Glance.Paris: OECD Publishing;2021. 10.1787/19991312

[2] Mayer S Berger M Konnopka A : In Search for Comparability: The PECUNIA Reference Unit Costs for Health and Social Care Services in Europe. *Int J Environ Res Public Health.* 2022;19(6):3500. 10.3390/ijerph19063500 35329189 PMC8948969

[3] Office of Health Economics: Scarce Resources in Health Care.London: OHE;1979;57(2):265–287. Reference Source 11665070

[4] de Meijer C Wouterse B Polder J : The effect of population aging on health expenditure growth: a critical review. *Eur J Ageing.* 2013;10(4):353–361. 10.1007/s10433-013-0280-x 28804308 PMC5549212

[5] Breyer F Lorenz N : The “red herring” after 20 years: ageing and health care expenditures. *Eur J Health Econ.* 2021;22(5):661–667. 10.1007/s10198-020-01203-x 32500244 PMC8214577

[6] Sleeman KE de Brito M Etkind S : The escalating global burden of serious health-related suffering: projections to 2060 by world regions, age groups, and health conditions. *Lancet Glob Health.* 2019;7(7):e883–e892. 10.1016/S2214-109X(19)30172-X 31129125 PMC6560023

[7] Aluttis C Bishaw T Frank MW : The workforce for health in a globalized context--global shortages and international migration. *Glob Health Action.* 2014;7(1):23611. 10.3402/gha.v7.23611 24560265 PMC3926986

[8] Drennan VM Ross F : Global nurse shortages-The facts, the impact and action for change. *Br Med Bull.* 2019;130(1):25–37. 10.1093/bmb/ldz014 31086957

[9] Pammolli F Riccaboni M Magazzini L : The sustainability of European health care systems: beyond income and aging. *Eur J Health Econ.* 2012;13(5):623–34. 10.1007/s10198-011-0337-8 21814838

[10] Organisation for Economic Co-operation and Development: Fiscal Sustainability of Health Systems Bridging Health and Finance Perspectives.Paris: OECD Publishing;2015. 10.1787/9789264233386-en

[11] Lorenzoni L Marino A Morgan D : Health Spending Projections to 2030.Paris,2019. 10.1787/5667F23D-EN

[12] Kane PM Daveson BA Ryan K : The need for palliative care in Ireland: a population-based estimate of palliative care using routine mortality data, inclusive of nonmalignant conditions. *J Pain Symptom Manage.* 2015;49(4):726–733.e1. 10.1016/j.jpainsymman.2014.09.011 25461670

[13] Keegan C Wren MA Walsh B : Projections of demand for healthcare in Ireland, 2015-2030: First report from the Hippocrates Model.Dublin: Economic and Social Research Institute;2017. 10.13140/RG.2.2.28563.58405

[14] Smith S Walsh B Wren MA : Geographic profile of healthcare needs and non-acute healthcare supply in Ireland.Dublin: ESRI;2019. 10.26504/rs90

[15] Department of Health: Health in Ireland Key Trends.Dublin,2022. Reference Source

[16] Organisation for Economic Co-operation and Development, Systems EOoH, Policies: Ireland: Country Profile.Paris,2017.

[17] May P Johnston BM Normand C : Population-based palliative care planning in Ireland: how many people will live and die with serious illness to 2046? [version 2; peer review: 2 approved]. *HRB Open Res.* 2020;2:35. 10.12688/hrbopenres.12975.2 32104781 PMC7017420

[18] Nolan A Ma Y Moore P : Changes in Public Healthcare Entitlement and Healthcare Utilisation among the Older Population in Ireland.Dublin: The Irish Longitudinal Study on Ageing;2016. Reference Source

[19] Department of Health: Sláintecare Action Plan.Dublin,2019. Reference Source

[20] Committee on the Future of Healthcare: Sláintecare Report.Dublin: Houses of the Oireachtas;2017.

[21] Thomas S Johnston B Barry S : Sláintecare implementation status in 2020: Limited progress with entitlement expansion. *Health Policy.* 2021;125(3):277–283. 10.1016/j.healthpol.2021.01.009 33531170 PMC9757858

[22] Department of Health: Sláintecare Progress Report 2021.Dublin, 2022. Reference Source

[23] Health Information and Quality Authority: Recommendations for Unique Health Identifiers for Healthcare Practitioners and Organisations.Dublin,2011. Reference Source

[24] Smith S Jiang J Normand C : Unit costs for non-acute care in Ireland 2016-2019 [version 1; peer review: 2 approved]. *HRB Open Res.* 2021;4:39. 10.12688/hrbopenres.13256.1 35317302 PMC8917322

[25] Gillespie P Carter L McIntosh C : Estimating the health-care costs of wound care in Ireland. *J Wound Care.* 2019;28(6):324–330. 10.12968/jowc.2019.28.6.324 31166856

[26] Donoghue OA McGarrigle CA Foley M : Cohort Profile Update: The Irish Longitudinal Study on Ageing (TILDA). *Int J Epidemiol.* 2018;47(5):1398–1398l. 10.1093/ije/dyy163 30124849

[27] May P De Looze C Feeney J : Do Mini-Mental State Examination and Montreal Cognitive Assessment predict high-cost health care users? A competing risks analysis in The Irish Longitudinal Study on Ageing. *Int J Geriatr Psychiatry.* 2022;37(7). 10.1002/gps.5766 35702991 PMC9328350

[28] May P Roe L McGarrigle CA : End-of-life experience for older adults in Ireland: results from the Irish longitudinal study on ageing (TILDA). *BMC Health Serv Res.* 2020;20(1):118. 10.1186/s12913-020-4978-0 32059722 PMC7023768

[29] Johnston BM : Progressing Sláintecare delivery from proposal to implementation: Insights from palliative care in Ireland. Awards Approved,2020. Reference Source

[30] Roe L : The Frail Brain and the Frail Body: Impact of FRAILty and COGnitive impairment on trajectories, patterns and costs in care in old age. Awards Approved,2018. Reference Source

[31] May P Johnston B Normand C : Palliative and End of Life Care in Ireland (PELCI) study. pelci.ie,2022.

[32] Health Research Board: Do we die as we live? Age socioeconomic status, healthcare utilisation and pathways to death in Ireland. Awards Approved,2017. Reference Source

[33] May P Normand C Matthews S : Projecting future health and service use among older people in Ireland: an overview of a dynamic microsimulation model in The Irish Longitudinal Study on Ageing (TILDA) [version 2; peer review: 2 approved]. *HRB Open Research.* 2022;5:21. 10.12688/hrbopenres.13525.2 36262382 PMC9554695

[34] Keegan C Brick A Bergin A : Projections of expenditure for public hospitals in Ireland, 2018-2035, based on the Hippocrates Model. *ESRI.* 2020. 10.26504/rs117

[35] Walsh B Keegan C Brick A : Projections of expenditure for primary, community and long-term care in Ireland, 2019-2035, based on the Hippocrates model. *The Economic and Social Research Institute and the Minister for Health.* 2021. 10.26504/rs126

[36] Briggs AH Claxton K Sculpher MJ : Decision modelling for health economic evaluation. Oxford: Oxford University Press;2006.

[37] Kearney PM Cronin H O'Regan C : Cohort Profile: The Irish Longitudinal Study on Ageing. *Int J Epidemiol.* 2011;40(4):877–84. 10.1093/ije/dyr116 21810894

[38] Brick A Normand C O'Hara S : Economic Evaluation of Palliative Care in Ireland: Final report. Report prepared for The Atlantic Philanthropies,2015. Reference Source

[39] Healthcare Pricing Office (HPO). HIPE Data. Accessed 16-01-2021,2021 Reference Source

[40] Central Statistics Office: Census 2011. 2012.

[41] Ward M May P Briggs R : Linking death registration and survey data: Procedures and cohort profile for The Irish Longitudinal Study on Ageing (TILDA). *HRB Open Res.* 2020;3:43. 10.12688/hrbopenres.13083.2 32789288 PMC7376615

[42] May P : Appendices to 'formal Health Care Costs Among Older People in Ireland: Methods and Estimates Using the Irish Longitudinal Study on Ageing (TILDA)'. *Open Science Framework* . [Dataset],2023. 10.17605/OSF.IO/76SYK PMC10565419 37829548

[43] Healthcare Pricing Office: ABF 2022 Admitted Patient Price List. Dublin: HPO;2022. Reference Source

[44] Central Statistics Office: Consumer Price Index. Accessed 2022-09-01,2022. Reference Source

[45] Health Information and Quality Authority: Guidelines for the Economic Evaluation of Health Technologies in Ireland. Dublin,2020. Reference Source

[46] Primary Care Reimbursement Service: Statistical Analysis of Claims and Payments 2020.Dublin: Health Service Executive;2020. Reference Source

[47] Moore PV Bennett K Normand C : Counting the time lived, the time left or illness? Age proximity to death, morbidity and prescribing expenditures. *Soc Sci Med.* 2017;184:1–14. 10.1016/j.socscimed.2017.04.038 28482276

[48] The Irish Longitudinal Study on Ageing. Computer-Assisted Personal Interview (CAPI): Wave 5.2018. Reference Source

[49] Personal Social Services Research Unit: Unit Costs of Health and Social Care 2019. Canterbury2019.

[50] Andersen RM : Revisiting the behavioral model and access to medical care: does it matter? *J Health Soc Behav.* 1995;36(1):1–10. 7738325

[51] Lawton MP Brody EM : Assessment of older people: self-maintaining and instrumental activities of daily living. *Gerontologist.* 1969;9(3):179–86. 5349366

[52] Callan T Colgan B Keane C : Modelling Eligibility for Medical Cards and GP Visit Cards: Methods and Baseline Results.Dublin: Economics and Social Research Institute;2015. Reference Source

[53] Stata Statistical Software: Release 15.[computer program]. College Station, TX: StataCorp LLC;2017. Reference Source

[54] R: A Language and Environment for Statistical Computing.[computer program]. Vienna, Austria: R Foundation for Statistical Computing;2022. Reference Source

[55] Deb P Norton EC Manning WG : Health Econometrics Using Stata.Stata Press;2017. Reference Source

[56] Connolly S Gillespie P O’Shea E : Estimating the economic and social costs of dementia in Ireland. *Dementia (London).* 2014;13(1):5-22. 10.1177/1471301212442453 24381036

[57] Pierce M Cahill S O’Shea E : Prevalence and Projections of Dementia in Ireland, 2011-2046.Dublin: Genio;2014. Reference Source

[58] Matthews S Pierce M O'Brien Green S : Dying and Death in Ireland: What Do We Routinely Measure, How Can We Improve?Dublin: Irish Hospice Foundation;2021. Reference Source

[59] Central Statistics Office: Births, Deaths and Marriages. 2021. Reference Source

[60] Ministers for Health announce biggest investment in Ireland’s Health and Social Care Services in history of the state.[press release]. Dublin,2022. Reference Source

[61] Central Statistics Office: Health Expenditure in Ireland 2019. System of Health Accounts 2019. 2020; Accessed 2023-02-01. Reference Source

[62] French EB McCauley J Aragon M : End-Of-Life Medical Spending In Last Twelve Months Of Life Is Lower Than Previously Reported. *Health Aff (Millwood).* 2017;36(7):1211–1217. 10.1377/hlthaff.2017.0174 28679807

[63] Raghupathi V Raghupathi W : The influence of education on health: an empirical assessment of OECD countries for the period 1995–2015. *Arch Public Health.* 2020;78(1):20. 10.1186/s13690-020-00402-5 32280462 PMC7133023

[64] Gaffney A Himmelstein DU Woolhandler S : Pricing Universal Health Care: How Much Would The Use Of Medical Care Rise? *Health Aff (Millwood).* 2021;40(1):105–112. 10.1377/hlthaff.2020.01715 33400569

[65] Connolly S Wren MA : Universal Health Care in Ireland—What Are the Prospects for Reform? *Health Syst Reform.* 2019;5(2):94–99. 10.1080/23288604.2018.1551700 30875264

[66] Irish Cancer Society: The Real Cost of Cancer.Dublin,2019. Reference Source

[67] Irish Heart Foundation: Pre-Budget Submission.Dublin,2019. Reference Source

[68] Tran PB Kazibwe J Nikolaidis GF : Costs of multimorbidity: a systematic review and meta-analyses. *BMC Med.* 2022;20(1):234. 10.1186/s12916-022-02427-9 35850686 PMC9295506

[69] Anderson E Durstine JL : Physical activity, exercise, and chronic diseases: A brief review. *Sports Med Health Sci.* 2019;1(1):3–10. 10.1016/j.smhs.2019.08.006 35782456 PMC9219321

[70] Donoghue O O'Connell M Kenny RA : Walking to Wellbeing: physical activity, social participation and psychological health in Irish adults aged 50 years and older.Dublin: Trinity College Dublin;2019. 10.38018/TildaRe.2016-00

[71] Hastings SN Whitson HE Sloane R : Using the past to predict the future: latent class analysis of patterns of health service use of older adults in the emergency department. *J Am Geriatr Soc.* 2014;62(4):711–5. 10.1111/jgs.12746 24635112 PMC3989455

[72] Shucksmith M Cameron S Merridew T : Urban-Rural Differences in Quality of Life across the European Union. *Reg Stud.* 2009;43(10):1275–1289. 10.1080/00343400802378750

[73] World Health Organization: Rural poverty and health systems in the WHO European Region.Copenhagen,2010. Reference Source

[74] Hosseinpoor AR Bergen N Barros AJD : Monitoring subnational regional inequalities in health: measurement approaches and challenges. *Int J Equity Health.* 2016;15(1):18. 10.1186/s12939-016-0307-y 26822991 PMC4730638

[75] Rodriguez Santana I Aragón MJ Rice N : Trends in and drivers of healthcare expenditure in the English NHS: a retrospective analysis. *Health Econ Rev.* 2020;10(1):20. 10.1186/s13561-020-00278-9 32607791 PMC7325682

[76] Whelan BJ Savva GM : Design and methodology of the Irish Longitudinal Study on Ageing. *J Am Geriatr Soc.* 2013;61 Suppl 2:S265–8. 10.1111/jgs.12199 23662718

