# Supplementary material for: Formal health care costs among older people in Ireland: methods and estimates using The Irish Longitudinal Study on Ageing (TILDA)
Source: HRB Open Res. 2023 Mar 4;6:16. [Version 1] doi: 10.12688/hrbopenres.13692.1 (PMC10565419; doi:10.12688/hrbopenres.13692.1)
Supplement: Peer review history

# Reviewer report: Reviewer response for version 1

**DOI:** 10.21956/hrbopenres.14977.r36274

**Published:** 2023-10-13

**Authors:** Sara Olofsson

**Affiliations:** 1 The Swedish Institute for Health Economics, Lund, Sweden

**Review of ”Formal health care costs among older people in Ireland: methods and estimates using the Irish Longitudinal Study on Ageing (TILDA)”**

 I would like to start by congratulating the authors on a well-performed and well-presented study. The work is of importance for policymakers as well as for future research. Overall, the study design seems well-constructed and outlined. It is easy to follow the authors' considerations behind the study approach and limitations are also well described. Additional material in the appendix (not reviewed) can make the result transparent and offer the reader additional insight. I believe that the manuscript is close to publishable as it is.

 However, below are some suggestions for clarification:

1. The study is based on a sample enrolled in interviews. It is well known that this might create a selection bias. The authors also adjust for this by applying a weighting approach. They also refer to appendices/other articles for more information. As a reader it would be informative to state/discuss in the manuscript (1) what characteristics the enrolled sample differs most from the general population, and (2) whether is there a risk that those enrolled could differ in cost due to unobservable characteristics and what would that imply?
2. Predictors of costs are analyzed using multivariate regression. In these kinds of studies, it is often difficult to identify the causes of costs as there are often confounding factors. The authors use careful language but draw some conclusions that indicate that associations are interpreted as causes. The manuscript could perhaps benefit from making it more clear to the reader that although there may be significant associations, this might not mean this should be interpreted as a cause of higher costs.
3. Finally, some notes on the terminology. Health care is often differentiated from caregiving (home, residential) as they usually serve different purposes. However, sometimes they are difficult to separate. Maybe a brief note or discussion of this could be added to the manuscript. Also, formal care differs from informal care provided by relatives/friends. Maybe I missed a clarification on these concepts, but if not, it would be beneficial to include them in the manuscript. Maybe it should also be highlighted in the discussion that informal caregiving could be substantial and that the true cost to society is therefore expected to be even higher.

Is the rationale for developing the new method (or application) clearly explained?

Yes

Is the description of the method technically sound?

Yes

Are the conclusions about the method and its performance adequately supported by the findings presented in the article?

Yes

If any results are presented, are all the source data underlying the results available to ensure full reproducibility?

Yes

Are sufficient details provided to allow replication of the method development and its use by others?

Yes

Reviewer Expertise:

Health economics

I confirm that I have read this submission and believe that I have an appropriate level of expertise to confirm that it is of an acceptable scientific standard.

# Reviewer report: Reviewer response for version 1

**DOI:** 10.21956/hrbopenres.14977.r36205

**Published:** 2023-10-10

**Authors:** Rachel Milte

**Affiliations:** 1 Flinders University, Adelaide, South Australia, Australia

Thank you for the opportunity to review this manuscript, which clearly reports this excellent piece of work. I find it makes a significant contribution to the literature in terms of healthcare costs among older people in Ireland, but also provides interesting findings making it of interest more broadly internationally. I find the data acquisition, preparation, and analysis and interpretation have all been undertaken with care and clearly a detailed understanding of the Irish health system and the strengths and limitations of the data available, which are acknowledged and at times potential recommendations to address these in the future are presented in the discussion (which is fantastic to see, so many papers miss out on providing concrete and practical ways their work can be built upon in the future). I have some minor comments which it would be good to address for clarity.

 Firstly, I find the use of the term multiple IADLs in the results strange - as I think you mean multiple impairments in IADLs as compared to having multiple IADLs. Secondly, I was unclear on the time to death variable less than 1 year - it seems this is solely based on whether they were part of the EOLI or CAPI interviews. But was there a chance that people in the EOLI interview lived for more than 1 year or than some people in the CAPI group died within 1 year? It seems that for the death within 2 years variable this was done by datalinkage - is there the option to do this for the 1 year variable and if so why was this not done? I think in this I am thinking about whether this variable represents the 'actual' costs associated with death, as compared to whether it is a cost associated with 'expecting death' in the next year? I would also like to see potentially trials of different models from the GLM family compared with the GLM with (these could be mentioned in the text and presented as an appendix) to check for the robustness of the results across different models. I find the discussion provides really clear and tangible implications for policy, and excellent suggestions for future research. I do hope that the authors go on and undertake some of this further research.

Is the rationale for developing the new method (or application) clearly explained?

Yes

Is the description of the method technically sound?

Yes

Are the conclusions about the method and its performance adequately supported by the findings presented in the article?

Yes

If any results are presented, are all the source data underlying the results available to ensure full reproducibility?

Yes

Are sufficient details provided to allow replication of the method development and its use by others?

Yes

Reviewer Expertise:

Dementia care, health economics, health services research

I confirm that I have read this submission and believe that I have an appropriate level of expertise to confirm that it is of an acceptable scientific standard.

# Reviewer report: Reviewer response for version 1

**DOI:** 10.21956/hrbopenres.14977.r36272

**Published:** 2023-10-03

**Authors:** John Mullahy

**Affiliations:** 1 University of Wisconsin-Madison, Madison, Wisconsin, USA

This is an exceptionally careful and well-executed paper studying questions that are hugely challenging yet important to answer. The authors have written a forthright manuscript that details lucidly what they have done—or, in some instances, have not done—and why. Many assumptions and decisions must be made in such a micro-costing exercise and the authors have gone to great pains to be clear about the nature of these assumptions and decisions. Micro-costing exercises will generally be an amalgam of art and science and the authors have succeeded in making clear where the science ends and the art (i.e. assumptions) begins.

 Given existing data it is difficult to imagine gleaning better insights into the nature and composition of healthcare costs among older people in Ireland than by learning from the results offered in this paper. The authors have evidently been excruciatingly careful in assembling their sample and have deployed what appear to be suitable empirical methods in their analyses.

 I would like to pose several questions, more in the spirit of clarification than criticism.

1. The data from the EOLI are intriguing and permit insights into healthcare resource use in older populations that are often not available in other surveys. In light of the magnitudes of the costs in the EOLI subsample I might hope that the authors could shed some further light on its nature in the Methods section. It would be interesting to know, for instance, how well or poorly the demographics of the decedents in EOLI sample correspond to those in the main sample before weighting (the weighted results appearing in table 4), with concerns about possible non-random selection into the EOLI sample being prominent.
2. Do the authors have access to any supplemental data that would help readers estimate the extent to which the costs associated with the omitted categories (table 2) are large or small or trivial as compared with costs in those categories that are included?
3. On page 6 appears: "Any sample-eligible CAPI or EOLI that was missing two or more of these four categories was flagged and removed from primary analysis as having insufficient outcome data. For those interviews missing one or fewer of these categories, and or missing any other categories of health care frequency, we imputed age- and sex-adjusted medians." In general I am not a fan of imputation methods and in this instance would be keen to know how the results presented in the paper would differ from those obtained if subjects having missing data for any category were deleted from the analytical sample.
4. I am curious to know why the authors (p. 7) elected to use a power-link in their GLM analysis rather than the more-standard log-link. They allude to "information criteria" but a bit more detail might be helpful.
5. On page 11 appears: "The composition of costs among non-zero users, across 20 quantiles of total health care costs in the pooled sample, is presented in Figure 5." Is this the only analysis in the paper that drops the zero-users or are there others? Either way the rationales for dropping zero-users in figure 5 might be made clearer.
6. Finally it is both obvious and defensible why the authors have undertaken a micro-costing analysis: Assigning currency values to different forms of healthcare utilization permits aggregation of the different categories of healthcare utilization. That being said I often suggest—and indeed am suggesting here—that in addition to their spending outcomes the authors also consider the merits of studying utilization data themselves as outcomes of potential interest. Such analyses would obviously need to be conducted category-by-category. But what such analyses can reveal is the extent to which variations in healthcare resource utilization are due to prices or quantities. This is perhaps a paper for another day but one whose merits might be given some thought.

Is the rationale for developing the new method (or application) clearly explained?

Yes

Is the description of the method technically sound?

Yes

Are the conclusions about the method and its performance adequately supported by the findings presented in the article?

Yes

If any results are presented, are all the source data underlying the results available to ensure full reproducibility?

No source data required

Are sufficient details provided to allow replication of the method development and its use by others?

Yes

Reviewer Expertise:

health economics

I confirm that I have read this submission and believe that I have an appropriate level of expertise to confirm that it is of an acceptable scientific standard.

# Reviewer report: Reviewer response for version 1

**DOI:** 10.21956/hrbopenres.14977.r33506

**Published:** 2023-04-25

**Authors:** Paddy Gillespie

**Affiliations:** 1 Health Economics & Policy Analysis Centre, National University of Ireland, Galway, Galway, Ireland

**Overview:**

 I would like to commend the authors on this excellent paper, which is the most comprehensive descriptive analysis of healthcare costs for older people that I have encountered in the Irish context. More generally, I believe that it makes a significant contribution to the literature relating to the nature, distribution, composition, and correlates of Irish healthcare costs. In particular, it covers a wide-ranging list of healthcare resources in its estimation of formal healthcare costs. This is far from a minor achievement given the historical paucity of available healthcare resource use and unit cost data in Ireland. The costing approaches applied to value each individual healthcare resource item appears to be pragmatically appropriate. In addition, the study includes an explicit consideration of proximity to death in its examination of the correlates of healthcare cost. This variable has been shown to be a significant driver of costs in the literature, but it is often omitted from applied papers of this kind given the difficulty in acquiring the data. To this end, the research team took on a number of additional and challenging lines of data collection, merging, and analysis, and the final paper is the richer for it. A minor criticism, which is related to these additional efforts in compiling the data, is that the paper does not flow as it might in places, but this is understandable given the need to describe the multiple methods employed. The authors employ appropriate statistical techniques for the multivariate analysis, although in places, they need to provide additional information. The extrapolation approach also appears to be appropriate. The authors clearly consider the limitations of their analysis, undertake sensitivity analysis, and highlight potential lines for future research. Finally, while the major contributions of the paper are descriptive in nature, this is not a flaw or limitation of the work. Indeed, this paper may prove to be a starting point in a future body of research that considers issues of causality as they relate to formal healthcare costs for older people in Ireland. This future work may be more impactful for policy and practice decision-making, but this paper provides a significant step forward. Congratulations to all of the team involved on the project.

 I have included a number of suggestions below that the authors may wish to consider, as I believe they will go to further enhance the paper. I look forward to reading the authors’ responses and to reviewing the next iteration of the paper.

*I should note that I was unable to access the appendices (which may well be a problem of my own making). However, I do not believe this is detrimental to my review and recommendation overall. I am aware that some of my suggestions may be addressed in the appendices.*

**Key Points**:

- ***Main Contribution***: The authors make a significant contribution in respect of the nature, distribution, composition and correlates of healthcare costs for older people in Ireland. This is clearly articulated in the paper.

 

- ***Main Multivariate Regression Result***: The authors highlight the both the *IADLs *and *proximity to death* findings as the key results from the multivariate regression analysis, given their economic and statistical significance. I wonder would the authors be better served by placing the emphasis on the *proximity to death* variable? My rationale is as follows, but I would be happy to hear a counter argument from the authors if they wish to make one.
- The authors adopt the Andersen framework to inform their choice of independent variables for the multivariate regression analysis. This approach, which categorises variables as *predisposing, enabling, need, *and *prior use, *is appropriate and the authors faced the not-insignificant challenge of selecting a list of variables from those available within TILDA to represent these categories. In particular, I would imagine the selection of variables to represent the ‘need’ category was challenging, and the authors went with a limited, informed and sensible set.
- The selection of IADLs variable proved to be an important one. This is interesting not just of its impact on the formal care system, but also for the potential and related impacts on the informal care system. That said, I do wonder if the inclusion of additional or alternative ‘need’ variables would impact on the economic and statistical significance of this variable? Indeed, the choice of GLM family and link function may also influence this outcome (see below for further comments). My suggestion would be to frame the IADLs finding in the context of the Andersen ‘need’ category: i.e. it indicates that ‘need’ factors appear to be strong predictors of healthcare costs and not to emphasise it individually as the key take-away.
- The authors also include a variable for *proximity to death*, and I think this is the key independent variable and should be interpreted as the key finding from the multivariate regression analysis. It proves to be economically and statistically significant, but it also highlights the importance of this variable in such costing studies, and the need for researchers to try to capture it.
- This is not to say the IADLs and other results should not be highlighted, but I think the focus on *proximity to death* may prove to be a cleaner and more robust foundation stone for the paper.    

 

- ***Summary Data for Healthcare Resource Usage and Individual Healthcare Costs***: As the authors suggest, their findings will be of interest to and use for future research projects. To this end, it would be helpful to report summary statistics (i.e. means, SDs, 95% Cis) for individual resource utilisation (e.g. GP, Hospital Inpatient Nights, OPD visits, etc.) and individual resource costs. These could be reported in the appendix (if they are not already).

 

- ***Descriptive and Distributional Analysis***: The authors present descriptive and distributional summary statistics of the total healthcare cost variable in tabular and graphical forms. As the authors suggest, these findings will be of interest to and use for future research projects. To this end, I think it would be valuable to include standard deviations and 95% confidence intervals for each of the mean cost estimates for the CAPI, EOLI, ALL samples in Table 5. The authors could also include 95% confidence intervals to Figure 2.

 

- ***Compositional Analysis***: The authors provide an informative graphical analysis of the distribution and composition of costs in Figures 3, 4 and 5. I wonder is there a way to include additional information to aid interpretation in the form of % of total figures for each decile (within or alongside the figures) of Figure 4, and € values for Figure 5? As mentioned, I could not access the appendix and the appendix may well be the best place for this data, but I think this is valuable information.

 

- ***Multivariate Regression Analysis***: The authors employ a GLM regression framework for the multivariate analyses. This is a standard and appropriate method for the statistical analysis of healthcare cost data, which can exhibit extreme skew and kurtosis in distribution. I think the authors need to provide additional information for the reader on the multivariate regression analysis (some of which may be presented in the appendix). In particular:
  - The authors should provide a rationale for the choice of GLM for the analysis of cost data.
  - GLM regression requires the selection of a ‘family’ and ‘link’ function. The authors used ‘information criteria’ to inform these choices. My understanding is that this approach is not quite correct. That is, the selection of ‘family’ is typically informed by a Modified Park test, and ‘link’ function by a combination of a Pearson correlation test, a Pregibon link test and a Modified Hosmer and Lemeshow test. I think that the authors should undertake these tests to make their ‘family’ and ‘link’ choices, and provide these results along with the regression results in Table 7 (or in the footnotes of Table 7).
  - The authors may wish to add further sensitivity analyses, which assume different ‘family’ and ‘link’ combinations, as results may be sensitive to this choice.
  - The approach adopted for the generation of standard errors in the regression analysis is also of interest. For example, given the nature of the TILDA dataset and how it is compiled, I wonder if there are issues relating to ‘clustering’ or ‘hierarchical data’ that need to be considered in the regression analysis? This information should be included and again, perhaps this is something that could be tested and explored in sensitivity analysis.

 

- ***Unconditional Quantile Regression***: Given that the authors rightly highlight the importance of the distribution of the estimated healthcare costs, and describe these results in the descriptive parts of the paper, I wonder if the authors considered conducting quantile regression to estimate and explore the associations between the independent variables and healthcare costs across the full distribution: e.g. deciles of the total healthcare cost distribution? While this would constitute additional analysis, I think it would provide complementary findings to those reported for research questions 1 and 2.
- ***Appendix:*** Please make these resources available

**Minor Points:**

**Abstract:**

- *Methods*: replace the term ‘main dataset’; use the term ‘2022 prices’;
- *Concussions:* align more closely to Conclusions section in the Discussion.

**Methods:**

- Table 2 and Figure 1 could be moved to the appendix.
- Provide more specific details on the imputation method employed (e.g. multiple imputation?).

**Results:**

- Update results to reflect suggested changes.
- Update tables to reflect suggested changes.
- Add further sensitivity analyses as suggested.

**Discussion:**

- Update as suggested: *proximity to death* variable should be the key variable of interest.
- The Andersen framework should be used to discuss the implications of the other regression findings.

Is the rationale for developing the new method (or application) clearly explained?

Yes

Is the description of the method technically sound?

Yes

Are the conclusions about the method and its performance adequately supported by the findings presented in the article?

Yes

If any results are presented, are all the source data underlying the results available to ensure full reproducibility?

No source data required

Are sufficient details provided to allow replication of the method development and its use by others?

Partly

Reviewer Expertise:

Health Economics

I confirm that I have read this submission and believe that I have an appropriate level of expertise to confirm that it is of an acceptable scientific standard, however I have significant reservations, as outlined above.
